# An Invertebrate Host to Study Fungal Infections, Mycotoxins and Antifungal Drugs: *Tenebrio molitor*

**DOI:** 10.3390/jof4040125

**Published:** 2018-11-12

**Authors:** Patrícia Canteri de Souza, Carla Custódio Caloni, Duncan Wilson, Ricardo Sergio Almeida

**Affiliations:** 1 Department of Microbiology, Center of Biological Science, State University of Londrina, Rodovia Celso Garcia Cid, Pr 445, Km 380, Londrina 86.057-970, Brazil; pcanteri@yahoo.com.br (P.C.d.S.); carlacalonic@gmail.com (C.C.C.); 2 Aberdeen Fungal Group, Institute of Medical Sciences, 4.15 ext. 7162, University of Aberdeen, Aberdeen AB24 3FX, Scotland; duncan.wilson@abdn.ac.uk

**Keywords:** alternative method of infection, *Candida* spp. *Cryptococcus* spp., invertebrate host model, mycotoxins, innate immunity, hemocytes, mealworm, tenecin, fungal infection

## Abstract

Faced with ethical conflict and social pressure, researchers have increasingly chosen to use alternative models over vertebrates in their research. Since the innate immune system is evolutionarily conserved in insects, the use of these animals in research is gaining ground. This review discusses *Tenebrio molitor* as a potential model host for the study of pathogenic fungi. Larvae of *T. molitor* are known as cereal pests and, in addition, are widely used as animal and human feed. A number of studies on mechanisms of the humoral system, especially in the synthesis of antimicrobial peptides, which have similar characteristics to vertebrates, have been performed. These studies demonstrate the potential of *T. molitor* larvae as a model host that can be used to study fungal virulence, mycotoxin effects, host immune responses to fungal infection, and the action of antifungal compounds.

## 1. Alternative Methods of Infection

Vertebrate animals, such as mice, are widely used in basic scientific and medical research for studies of microbial pathogenicity, testing of substances of pharmacological interest, the production of vaccines, evaluation of the effectiveness of antibiotics, and in the development of new therapies [1,2]. However, the pain, distress, and sacrifice of animals during scientific experiments has been questioned by researchers and society [3]. Thus, the ethics of this practice began to be reformulated since the nineteenth century [4], which determined the approval of laws and actions to control the use of animals in research.

In addition to ethical concerns, the use of animals in research requires a high economic investment, skilled labor, and time-consuming protocols [5,6]. In this context, several industrial sectors and regulators of product quality are increasingly being pressured to replace, or reduce, animal experimentation with alternative methodologies. Alternative proposals are based on the principle of the 3Rs, which was introduced to the scientific community in 1959 by Willian Russell and Rex Burch in the book Principles of Humane Experimental Technique [5]. The acronym stands for “Refinement”, which proposes to better plan the procedures performed on animals, with the aim of minimizing pain and/or stress; “Reduction” of the number of animals in the experiments, using alternative techniques to obtain reliable results; and “Replacement”, which advocates the replacement of vertebrate use by non-sentient animals. Indeed, the use of invertebrate animals as alternative model hosts has increased dramatically in the last decade, in order to avoid and/or reduce the use of vertebrates in experiments [6].

Insects have been increasingly chosen as a model host to study microbial infections [7]. This is due to the presence of an innate immune system similar to vertebrates. Although the responses of the insect’s immune system are not specific, because they do not produce antibodies, they possess a complex innate immune system with both cellular and humoral elements [8,9]. The innate humoral response of insects is orchestrated by processes that inhibit the proliferation of pathogens and assist in their elimination, such as melanization, hemolymph coagulation, induction of the synthesis of reactive species, and antimicrobial peptides [10,11]. Cellular responses are mediated by cells called hemocytes, which play an important role in defense, as they are involved in phagocytosis, nodule formation, and encapsulation [12].

The use of insects as an alternative model also provides other advantages, such as the absence of ethical restriction. In addition, when compared to traditional mammalian models, breeding is inexpensive and easy to maintain, as it does not require a sophisticated laboratory. In addition, the lifecycle is short, allowing large-scale experiments in a short period of time. Thus, several insects have been widely used, notably, the fruit fly *Drosophila melanogaster*, and some species of Lepidoptera and Coleoptera [13,14].

Here, we will discuss the use of *Tenebrio molitor* larvae (mealworm) as an alternative host model in the study of pathogenic fungi, including the biological characteristics of the animal, particularities regarding its immunity, as well as the first studies involving medically important fungi.

## 2. *Tenebrio molitor*

**Figure 1:**
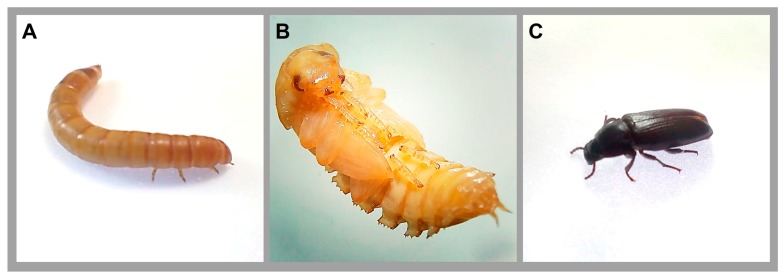
Lifecycle of the beetle *Tenebrio molitor*: (**A**) larval stage, (**B**) pupal stage, and (**C**) adult stage.

*Tenebrio molitor* (Linnaeus, 1758), popularly known as the mealworm, belongs to the order Coleoptera and family Tenebrionidae. It is found worldwide, however, it has climatic preferences for temperate regions of the northern hemisphere [15,16]. This insect exhibits holometabolism (complete metamorphosis), in which its lifecycle is composed of 4 stages: embryo stage (eggs), larval phase, pupa phase, and adult phase (Figure 1). In addition, the larval phase presents a series of stages or instars, which is reached after the ecdysis process [15,16]. According to Park et al. [17], *T. molitor* presents up to 20 instars.

Normally, the larval phase occurs during spring, which is the pupation season. At the beginning of summer, beetles begin to appear, which represent the adult phase of the insect [18]. However, lifecycle stages may increase or decrease depending on environmental conditions. A number of studies have demonstrated that the number of instars of *T. molitor* varies in response to different factors [19], such as temperature [20,21], humidity [22], oxygen concentration [23], quality of nutrients [24], and populational density [25].

The feeding of the larvae is based on cereals or milled grains. Thus, this insect is considered to be a pest of stored grain and bran [26,27]. Larvae are also considered an excellent source of animal protein, and are used as feed [28] for several domestic or zoo animals, including fish [29], lizards [30], birds [31], and bats [32]. In many countries, these larvae are also used for human consumption [33,34].

## 3. Cellular and Humoral Immunology of *T. molitor* as a Tool to Study Human Immunity

**Figure 2:**
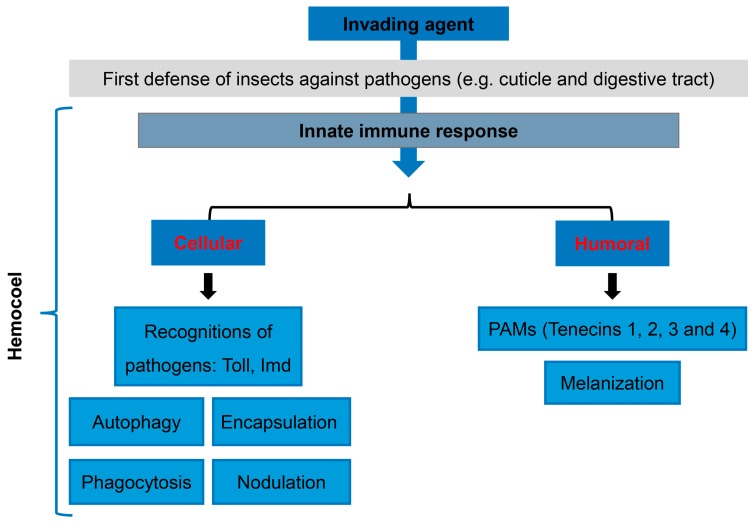
Schematic diagram of *T. molitor* immune response to pathogenic fungi.

In order to protect against potentially pathogenic microbes, the integument and digestive tract represent the first line of defense, which plays an important role against infections. However, when these barriers are overcome, the invading pathogen is exposed to a range of cellular and humoral mechanisms (Figure 2) [35].

**Figure 3:**
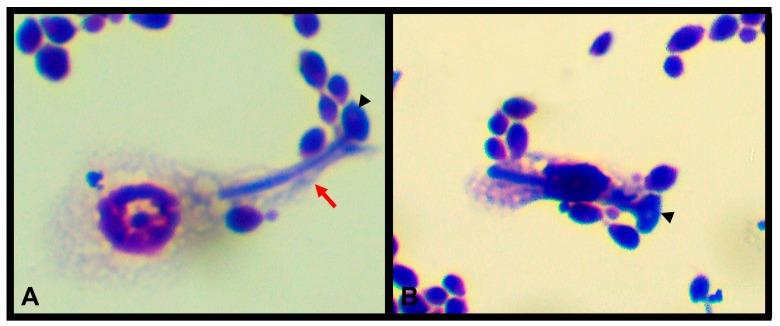
Plasmatocyte (**A**) and granulocyte (**B**) phagocytizing *Candida albicans*. May-GrünwaldGiemsa staining and 2 h of co-incubation. Cytoplasmic projections (pseudopodium—red arrow) towards the fungus (arrowhead) is observed. 100× objective lens.

In a study carried out by Urbański and collaborators [36], four hemocyte types were observed: granulocytes, plasmatocytes, oenocytoids, and prohemocytes, according to their size and morphology. Prohemocytes have been considered to be stem cells, with the ability to differentiate into other cell types [12]. In addition, granulocytes and plasmatocytes are the most abundant types in hemolymph, the only ones with adherent properties, and are the cells responsible for phagocytic activity (Figure 3), encapsulation, and nodulation [37,38]. The enzyme phenoloxidase (PO) plays a key role in mediating this important defense mechanism. This enzyme is the main component responsible for the melanization process of hemocytes attached to the pathogen [39]. Studies indicate that prophenoloxidase (proPO) is predominantly synthesized by granulocyte and oenocytoid hemocytes, and depends on the insect species [40].

The process of phagocytosis refers to the internalization of foreign bodies by a single hemocyte through the formation of pseudopods (Figure 3) [41]. The similarity of phagocytic activity between hemocytes and neutrophils in vertebrates has been noted [42]. In addition, autophagy, which is another evolutionary mechanism conserved in insects, has already been demonstrated in *T. molitor* [43]. This autophagic response assists in cellular homeostasis, and also plays an important role in cellular innate immune responses because it contributes to the phagocytic elimination of pathogens [41,43,44].

When the pathogen burden is too large to be phagocytosed, nodulation and encapsulation processes occur. These mechanisms, unlike phagocytosis, refer to the multiple actions of hemocytes, which aggregate around the target [12]. These processes are complex and exhibit cellular and humoral immune reactions, including activators of a complex enzyme system, termed prophenoloxidase (proPO) [45]. In the process of melanization, the isolation of pathogens usually occurs through encapsulation. In addition, the microorganisms that are involved in a melanized layer of hemocytes die from lack of oxygen or from the release of compounds produced by phenoloxidase activity [46], such as reactive oxygen species [11]. It has been demonstrated that melanin is crucial for *T. molitor* immunity, including cuticular melanization, which results in a resistance to pathogen infection [47]. Barnes and Siva-Jothy [48] observed that larvae with higher degrees of cuticular melanization showed greater resistance when exposed to the entomopathogenic fungus *Metarhizium anisopliae*.

Beyond melanization, the synthesis of peptides with antimicrobial activity (PAMs), is one of the main mechanisms of humoral immunity against pathogens, both in insects and vertebrates [49,50]. In *T. molitor*, 4 different types of PAMs are known: tenecins 1, 2, 3, and 4. Tenecin 2, which acts against Gram-negative bacteria and fungi, is a molecule similar to coleoptericin and holotricin, which are PAMs produced by the coleopterae *Zophobas atratus* and *Holotrichia diomphalia*, respectively [51,52]. Tenecin 3 is a glycine-rich peptide which targets fungi [53,54,55,56], whereas tenecins 1 and 4 have activity only against Gram-positive and Gram-negative bacteria, respectively [56,57].

Immunogenic molecules of microorganisms, such as fungi β-1,3-glucan, or lipopolysaccharides of Gram-negative bacteria and peptidoglycans of Gram-positive bacteria, can induce the synthesis of PAMs in insects through the activation of two main signaling pathways: the Toll-like pathway and the immunodeficiency pathway (Imd) [56,58]. Toll-like membrane receptors are an evolutionarily conserved receptor family, and the Imd pathway is analogous to the tumor necrosis factor (TNF) signaling pathway in humans [59]. It is known that tenecins 1 and 2 are activated mainly by the Toll signaling pathway [52]. Tenecin 4 is activated by both the Toll pathway and the Imd pathway [56]. Tenecin 3, on the other hand, is constitutively produced (without the need for induction) and the molecular mechanisms involved in its synthesis have not yet been determined [56,60].

In addition, a recent study has discovered a novel membrane receptor produced by *T. molitor*. This receptor is a class C hijacker, which plays a key role in phagocytosis of fungi and Gram-positive and Gram-negative bacteria [61].

Although insects do not have an adaptive immune response, studies with *T. molitor* larvae and entomopathogenic fungi have demonstrated that the innate immune response of this insect can be long-lasting, even when the immunogenic agent no longer present. The process has been termed as immune priming, and is known to provide a prophylactic effect, being functionally similar to the adaptive immune response of vertebrates [62]. For example, a previous exposure of larvae to a nylon monofilament increased insect protection when challenged by *Beauveria bassiana* after a week after the first exposure. The material injected into the animal apparently induced the encapsulation response, indicated by darkening of the body of the insect due to melanization [63]. Another study showed that larvae that were pre-challenged with lipopolysaccharides also presented greater resistance to *M. anisopliae* infection [62].

Another important attribute related to immune priming is its species-specific character. Larvae that were exposed two times to the same pathogen (*M. anisopliae*) presented a higher survival rate than larvae that were challenged with this fungus and later exposed to *Bacillus thuringiensis* and *Serratia marcescens* [64]. In addition, immune priming has the ability to be transmitted between generations. Immune-challenged mothers can improve their offspring immunity through transgenerational immune priming. It has been proven that enhancement of offspring immunity increases egg hatching success and larval survival [65].

Thus, despite the lack of a true adaptive immune system, *T. molitor* exhibits complex immune responses with evidence of recall, demonstrating its potential as an alternative infection model.

## 4. *T. molitor* as an Alternative Host in the Study of Human Pathogenic Fungi

When compared to other alternative host models used in the study of human fungal pathogens, *T. molitor* has some major advantages. The mealworm can be incubated at 37 °C which, for many pathogens, is a critical environmental cue for the expression of virulence factors. For example, physiological temperature stimulates the production of invasive hyphal filaments by *C. albicans*. Other models, however, do not support such high temperatures. *D. melanogaster* survives at a temperature of 18 °C to 30 °C, and the best temperature to perform tests with the *Caenorhabditis elegans* worm is 15 °C to 25 °C [66].

**Figure 4:**
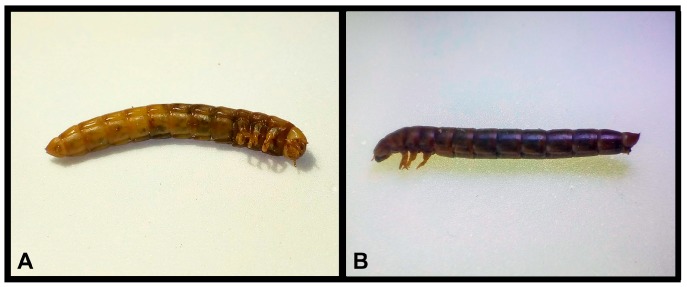
*T. molitor* larva infected with 106 *C. albicans* yeasts. (**A**) 24 h after infection. (**B**) 30 h after infection.

A second important feature is the size of the larva. Since *T. molitor* larvae are relatively large, this allows the extraction of a considerable volume of hemolymph (5–10 μL/larva of the 12th instar) to analyze. This represents an advantage over other model organisms, such as the fruit fly, since its size allows the extraction of only small volumes of hemolymph (0.05–0.3 μL/larva of 3rd instar) [67]. Another advantage is that the inoculum is administered by injection, allowing direct introduction into the hemolymph of the animal [68]. Additionally, dead larvae are easily identified because they turn brown through melanization, as illustrated in Figure 4.

Another widely used model is the wax moth larva *Galleria mellonella*. This insect has similar characteristics as *T. molitor* as an alternative host, since it can be incubated at 37 °C, the inoculum can be supplied by injection [66], and it also has a substantial volume of hemolymph. However, its breeding in laboratory conditions is more laborious, and *G. mellonella* is not marketed in some countries, forcing institutions to maintain their own supply. This raises the costs of the experiments, and requires the technical manpower responsible for their maintenance. In Brazil, there are several commercial suppliers of *T. molitor*.

## 5. Investigating Physiological Changes Due to Ingestion of Mycotoxins

In 1973, Reiss [69] published the first work involving the use of *T. molitor* in the investigation of diseases caused by fungi. Beetle larvae were used as an animal model to understand the physiological changes that occurred after ingestion of food contaminated with mycotoxin-producing fungi. In this study, the larvae were fed with bread contaminated with *Aspergillus niger*, *Aspergillus flavus*, *Penicillium expansum*, *Cladosporium herbarum*, and *Neurospora sitophila*, and their masses were measured daily. It was observed that all isolates, except for *N. sitophila*, presented toxicity. *A. flavus* had the greatest inhibitory effect on larval development. These findings demonstrated the similarity of the physiological response of the insect to mammals, since exposure to aflatoxin (FA), produced by *A. flavus*, is associated with weight loss and reduced stature in children [70,71]. In addition, it is also related to economic losses due to the reduction of the body mass of pigs and cattle [72,73,74].

Similarly, insect feeding with cereals colonized by species of the genus *Fusarium* also leads to a reduction of growth [75], and even death of the larvae [76]. These findings, again, correlate with research on vertebrates. Fumonisin (FM), the mycotoxin produced by *Fusarium* spp., is associated with reduced food consumption and weight in pigs [77]. In addition, Kimanya et al. [78] showed that children who ingested FM in more than the maximum provisional daily dose, grew less and were lighter than children who did not exceed this limit.

On the other hand, Bosh et al. (2017) demonstrated that *T. molitor* larvae fed with an aflatoxin B1-contaminated diet did not present alterations in survival rate and body weight, nor was there an accumulation of the substance in the animal [79]. An explanation for these findings is based on the ability of fungi to produce different types of mycotoxins. In the studies cited above, the fungus was mixed into the diet, and was then heat inactivated, leaving the mycotoxins secreted by it in the samples [69,70,71,72,73,74,75,76,77,78]. Thus, these findings suggest that their toxic effects are due to a combination of toxins, rather than the action of an isolated mycotoxin.

## 6. Studying Infections Caused by Fungi of Clinical Interest and Evaluating the Antifungal Activity of Compounds

Yeasts are the primary fungi isolated from disseminated infections, with *Candida* and *Cryptococcus* species being the most frequently isolated. Both of these species are dimorphic, however, *C. albicans* hyphae have the invasive morphology and are associated with virulence, whilst *Cryptococcus* grow as yeast during infection [80,81,82,83]. In this context, our research group showed, for the first time, the utility of *T. molitor* as a model host to study infections caused by *Candida albicans* and *Cryptococcus neoformans* [84]. In this work, *T. molitor* larvae were infected with different concentrations of *C. albicans* and *C. neoformans*, and the mortality was determined. We found that the larval survival decreased proportionally to the increase in inoculum concentration [84], similar to that of *G. mellonella* and mice when challenged with these yeasts [85].

In addition, histological sections of the infected tissue demonstrated the formation of *C. albicans* hyphae and mycelia [84], similar to what is observed in other models, including mice [86], hemoperfused porcine liver [87], chicken chorioallantoic membrane [88], and *G. mellonella* [85]. In the tissue infected with *C. neoformans*, the pathogenic yeast morphology was observed, as in *Galleria* and mice models, and clinically [85,89].

This insect was also recently chosen to study the virulence of *Malassezia furfur*. It was shown that yeasts cultivated under conditions of nutrients scarcity were more virulent to *T. molitor* larva than those cultivated in complete medium [90]. Nutritional stress leads to a change in microorganism metabolism which, in order to adapt to these conditions, can express their virulence factors [91]. The authors obtained similar results using *C. elegans* as an animal model and concluded that, in this sense, the methodology using *T. molitor* was efficient [90].

*T. molitor* can also be used to study infections caused by filamentous fungi. [92] infected *T. molitor* larvae with *Fonsecaea* species (etiological agents responsible for human chromoblastomycosis, a granulomatous and chronic skin infection) [93] and found structures similar to muriform cells in the tissue infected with *Fonsecaea pedrosoi*. In an earlier study performed with *G. mellonella*, this event was not observed [94]. However, conidia of *F. pedrosoi* presenting this morphology are common in histological samples of mice previously infected by the fungus [95,96].

Given the utility of the mealworm as an alternative model in the evaluation of different anthelmintic compounds [97], Morey and collaborators [98] used this model to verify the efficacy of a potential antifungal agent. In this work, the larvae of *T. molitor* were infected with *Candida tropicalis*, and treated with a tannin-rich fraction from a *Stryphnodendron adstringens* extract. The survival of the insects that received the inoculum containing the fraction of the plant extract was significantly higher compared to the group without treatment, indicating not only that the larvae are sensitive to *C. tropicalis* infection, but can also be used as an alternative host model to evaluate the activity of potential antifungal compounds [98].

## 7. Conclusions

*T. molitor* has a complex innate immune system, consisting of humoral and cellular responses and, because it is sensitive to infection with a range of medically important microorganisms, its use as an alternative host in the study of pathogenic fungi is now being recognized. As discussed here, *T. molitor* represents a potential invertebrate host that could be used to study the virulence of fungi, toxicity due to the ingestion of mycotoxin-producing fungi, the host’s immune responses to fungal infections, and the action of antifungal compounds. However, the current number of studies is limited. In addition, there is a need to standardize the tests using *T. molitor* larvae in the study of pathogenic fungi.
